# Contrasting real time quantitative measures (weekly SMS) to patients’ retrospective appraisal of their one-year’s course of low back pain; a probing mixed-methods study

**DOI:** 10.1186/s12998-018-0222-y

**Published:** 2019-02-26

**Authors:** Lise Hestbaek, Cornelius Myburgh, Henrik Hein Lauridsen, Eleanor Boyle, Alice Kongsted

**Affiliations:** 1 Department of Sports Science and Clinical Biomechanics, University of Southern Denmark, Odense, Denmark; 2 Nordic Institute of Chiropractic and Clinical Biomechanics, Campusvej 55, 5230 Odense M, Denmark

**Keywords:** Back pain, Course, Trajectories, Recall, Mixed methods, SMS, Interview

## Abstract

**Background:**

Due to the recurrent nature of low back pain (LBP), the traditional concepts of cure and recovery are challenged, and investigating the course rather than status at fixed time-points may help us understand prognosis as well as treatment effect. However, methods of frequent measuring still need development and validation. Therefore, this study aims to evaluate the agreement between continuous, quantitative self-assessment (weekly SMS) of the course of LBP over a one-year period and qualitatively derived retrospective patient self-appraisal of the same time-period.

**Methods:**

Participants were 32 subjects with LBP from primary care. The quantitative measures consisted of weekly SMS questions for one-year about pain intensity, days with LBP, and activity limitations for that week. For each subject, the weekly responses were graphed and categorized into categories based on intensity, variation and overall change patterns. Qualitative measures were based on semi-structured telephone interviews one-year after a consultation for LBP, where two coders independently categorized the self-appraisal of LBP course into the same predefined categories as the SMS-based trajectories. Furthermore, patients’ perceived overall recovery was related to variation patterns from SMS track.

**Results:**

There was perfect agreement for 48% in the pain intensity domain, 53% in the variation domain and 63% in the change pattern domain. Most of the discordant cases were classified in neighboring categories with the majority relating to fluctuating patterns. The self-perceived overall recovery status seemed to be reflected quite well by the quantitative measures of pain intensity and days with pain in this study.

**Conclusion:**

This study shows that a real time quantitative measure (weekly SMS) and the patient’s retrospective appraisal do not fundamentally differ in their reflection of the one-year course of LBP.

As a first investigation into this area, these results are promising, as longitudinal quantitatively derived trajectories of LBP seem to reflect the lived experience of the patient to a large degree. Furthermore, the patient’s ability to retrospectively recall their one-year course of LBP appears to be quite good. Future studies should focus on refining the categories of trajectories.

**Electronic supplementary material:**

The online version of this article (10.1186/s12998-018-0222-y) contains supplementary material, which is available to authorized users.

## Background

The assessment of patients’ outcomes is essential in all areas of health care as well as in clinical research. However, in diseases that are not life threatening it is not straight forward defining what a “successful” outcome is. In back pain, this is clearly demonstrated in two reviews of recovery definitions [1, 2]. In the latter, Kamper et al. identified 66 different outcome measures used in 82 studies related to low back pain (LBP) [2]. The different measurements primarily cover various definitions of pain, disability, and to a lesser extent physical performance, overall recovery and return to work; alone or in combination [2]. It is self-evident that such absence of a clear conceptual understanding of “success” is not only a barrier for understanding the effect of interventions, but also for the development of accurate and relevant outcome measurements.

Qualitative studies have demonstrated that patients’ sense of recovery from LBP is both complex and highly individualized [3], but nevertheless the overarching themes support the constructs used in quantitative research, namely pain and disability [4–6]. However, many other factors probably influence the impact of LBP on the patients’ health, participation in society and quality of life, and thus their overall outcome [3, 7–9]. Equally important as the concept measured, is the time frame across which the concept is measured. LBP often presents as a recurrent condition characterized by fluctuating patterns rather than a finite condition-related resolution [10, 11]. Summarizing pain over time is a complex cognitive process and it seems to be influenced by both the physical and mental state of the patient on the day of questioning [12], and therefore a single measure at a predefined time-point is unlikely to capture the experience of LBP well. This introduces a large degree of uncertainty, which has not been appropriately addressed in LBP research [11]. It may also explain some of the apparent discrepancy between standard quantitative outcome measures, such as pain intensity or sick leave, and self-appraised recovery that has been reported previously [13, 14].

In attempts to more accurately reflect the course of LBP rather than using a single time-point measure, trajectories of LBP based on frequently repeated measures have been created [15–18]. These studies have demonstrated the existence of distinct clinical course patterns of LBP which would not have been revealed by measuring outcome at only one or a few points in time, by summarizing individual trajectories into a summary score, or by population means in longitudinal analyses [15]. However, descriptions of these trajectories are still being developed and the interpretation is therefore difficult. For instance, the level of details in the trajectories, and thus the number of resultant subgroups, varies from four [16] to twelve [15] distinct trajectories in LBP patients from primary care, when using different analytical strategies. Furthermore, it is unknown how large fluctuations should be, before they can be considered to be above measurement error and thereby relevant for clinical interpretation [19]. An attempt to operationalize the different trajectories has been made by Kongsted et al. by combining results from 10 different cohorts investigating data-driven SMS-based trajectories [19]. This has resulted in descriptions of trajectories that can be applied across datasets [20]. These descriptions are based on three domains: pain intensity, variation and overall change pattern across the observed period. However, these trajectories are data driven based on very simple questions, and they have not been validated against patients’ subjective experiences. Therefore, we need to understand if these categorizations reflect differences between trajectories that are important for the patients’ perception of their course of pain. To do this, we take advantage of previously collected data to compare patients’ recollections of their pain to SMS-based trajectories.

### Aims

The primary objective of this study was to compare SMS-based trajectories as defined by Kongsted et al. [19] to interview-based trajectories derived from patients’ 1 year recall of their LBP experiences after a consultation for LBP. Secondly, we compared the SMS-based trajectories to peoples’ overall assessment of their perceived recovery based on interviews.

## Methods

### Design

A probing convergent mixed method study comparing quantitatively and qualitatively derived outcome measures in a primary care setting [21].

### Setting and participants

Participants were recruited from a prospective cohort study with one-year follow-up*.* The study included LBP patients from general physician practices and chiropractic practices to reflect LBP-patients in Danish primary healthcare.

Chiropractors from 17 out of 21 invited chiropractic clinics from the research network of the Nordic Institute for Chiropractic and Clinical Biomechanics agreed to recruit consecutive patients with LBP from September 2010 to January 2012. Prior to inclusion, patients received written and verbal information about the study. During the 16 months, 947 patients were included.

All 800 GPs in the Region of Southern Denmark were invited to participate in a quality development initiative by the Audit Project Odense [22]. The objectives of this initiative were to evaluate the use of the STarT Back Tool, implementation of electronic data capture and the establishment of a cohort of patients with LBP to be followed prospectively. Eighty-eight general practitioners agreed to participate. During 10 weeks in 2011, they registered 421 patients who consulted for LBP. Following the consultation, the patients received an envelope containing information about the prospective study described above, an invitation to participate, a baseline questionnaire and a prepaid return-envelope. The baseline questionnaire was returned by 206 patients. The cohort study has been described in detail previously [23, 24].

The inclusion criteria for both chiropractic and general practice populations were LBP with or without radiating pain, 18–65 years of age, and access to a mobile phone. Exclusion criteria were pregnancy, suspicion of serious pathology and inability to read and write Danish. Furthermore, an additional exclusion criterion for chiropractic practice was having had more than one health care visit for LBP within the last 3 months.

For this study, we drew our sample from the participants who had completed the one-year follow-up in the cohort study and no longer received the SMS questions. To ensure a broad and inclusive set of responses we employed a purposive maximum variation sampling framework: The first 12 participants were consecutively included at least a week after they completed their 1-year follow-up, and were thus eligible for inclusion. Three attempts were made to contact a particular individual (on different weekdays). If the individual could not be contacted, they were excluded and the next person on the list was contacted for potential inclusion. These 12 participants were supplemented by a maximum variation sample of 20 respondents. These were identified by distinctly different SMS-based trajectories and ensured a variation in the course patterns and has been described in detail previously [9]. Patients were excluded if they answered the SMS-questions less than 26 of the 52 weeks.

### Quantitative data collection

All members of the cohort study were sent weekly SMS questions for 1 year. The cohort members first responded to the following question “How many days did you have low back pain during the last week? (A number between 0 and 7)”. If they responded with at least 1 day of LBP, they were subsequently asked the following questions: “How intense was the pain typically on a scale from 0 to 10?” (referred to as Numeric Rating Scale (NRS)) and “How many days during the past week has your low back pain limited your activities? (A number between 0 and 7)”. If zero LBP days were reported, the NRS and activity limitation were assumed to be negligible and thus coded as zero. If cohort members did not respond to the SMS-questions for two consecutive weeks, a research assistant telephoned them to remind them about the study.

### Qualitative data collection

We conducted semi-structured, telephonic interviews. Telephonic, rather than face-to-face interviews were chosen due to a wide geographic spread of respondents in the cohort study. The interview guide consisted of three core questions: “Are your back problems over?”, “To what extent have your back problems affected you?” and “Has anything special occurred during the last 12 months in relation to your low back problems?” Thus, interviewers avoided drawing attention to specific domains related to the problems, i.e. pain intensity. Participants were encouraged to explain and elaborate their answers.

### Analyses

**Fig. 1:**
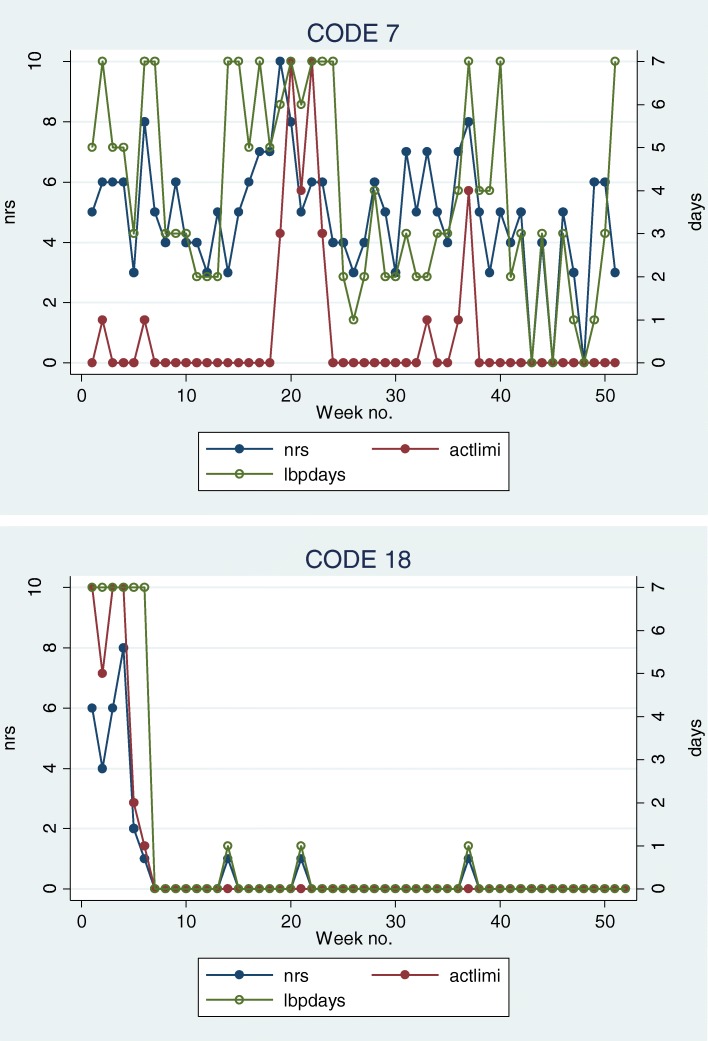
Examples of SMS trajectories based on weekly SMS questions to LBP patients in primary care. The code refers to the identifier in Additional 1

**Fig. 2:**
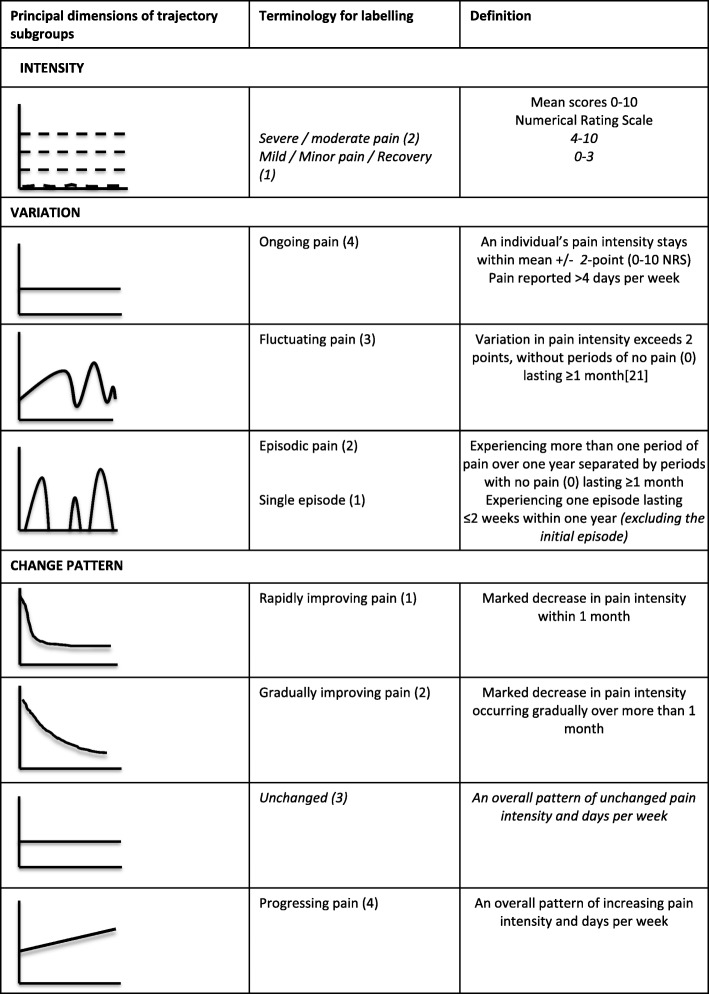
Trajectory definitions as defined by Kongsted et al., modified for the present study to include the initial episode. The pain intensity has been changed from four to two episodes, and the category for the ‘unchanged’ pain pattern has been added. Modifications indicated in italics. The numbers in parentheses after the labelling refer to the numbers in Additional file 1

SMS trajectories were illustrated in time-series line plots for the 32 interviewed subjects. Two examples are shown in Fig. 1. Based on the intensity and frequency questions in these plots, the one-year SMS-based trajectories of the interviewed individuals were independently categorized by two authors (HHL and LH) in accordance to the predefined categories for each of the three domains (pain intensity, variation and change patterns). This was done by following the operational criteria from Kongsted et al. [19]. However, in the formation of these criteria, the first 9 weeks after an initial consultation for LBP were ignored in order to describe a period of clinical stability. Therefore, we modified the criteria to accommodate the initial episode by adding *‘excluding the initial episode’* to the criteria for the ‘single episode’-category. Furthermore, a category describing the change pattern as ‘unchanged’ was added and the four categories in the intensity domain were combined into two categories (‘none to mild’= NRS 0–3 and ‘moderate to severe’: NRS 4–10), because we considered that to be a more realistic level of distinction obtained from interviews. The categories and their operational criteria are shown in Fig. 2, and modifications from the original operational criteria are indicated in italics. The two authors met to discuss the categorization and discrepancies were resolved by consensus.

Interviews data were recorded, transcribed verbatim and translated into English, since both assessors were native English speakers (EB and CM). Using the operational criteria, a codebook was derived by CM. Codes were applied for each of the categories within the three domains shown in Fig. 2, and the frequency of appearance for each code was noted. In some cases, one or more of the domains could not be classified because the issue was not mentioned, and these were consequently registered as missing. Typical quotes were chosen to substantiate the choice of trajectory within the three domains. Responses to the question “*Are your back problems over?*” were coded as ‘yes’, ‘no’ or ‘unsure’. Two of the authors (EB and CM) independently coded each interview and met to discuss the disagreements.

First, the interview-based trajectories and the SMS-based trajectory categorizations were cross-tabulated separately for the three domains and the percent agreement was determined. Next, cases with disagreement were described with respect to the SMS-track. For this description, SMS questions about activity were also considered as potential explanations since the SMS categorizations were only based on questions about pain intensity and frequency.

Weighted Kappa statistics were intended but due to several empty cells in the tables, this was not feasible.

For the secondary objective, to compare the SMS-based trajectories to peoples’ overall assessment of their perceived recovery based on interviews, we compared and contrasted the response to the interview question “*Are your back problems over?*” with the SMS-based trajectories from the ‘variation’ domain.

### Post-hoc analysis

It was noted that more patients were categorized as ‘moderate to severe’ with respect to pain intensity in the interviews than in the SMS-based trajectories, and that many of these disagreements related to patients who had been pain free for part of the time. Therefore, categorization based on SMS-track was repeated with the mean pain score based only on the intensity of pain during pain episodes, i.e. pain free episodes ignored.

**Fig. 3:**
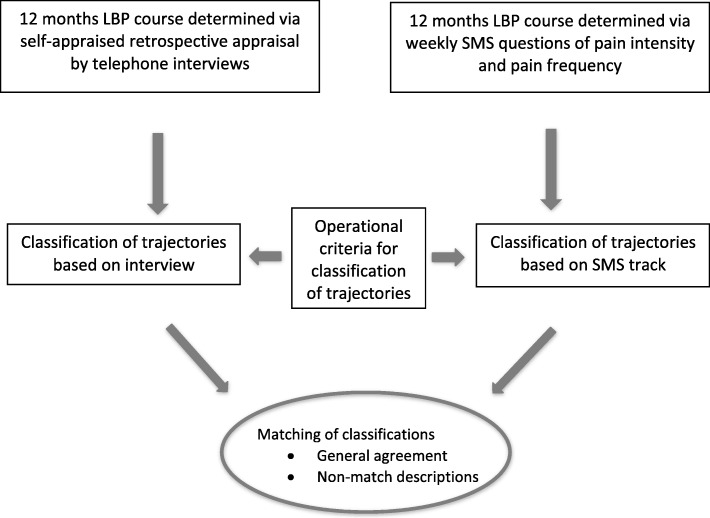
Study flow diagram

The flow of the study is illustrated in Fig. 3.

## Results

### Participants

SMS trajectories from two subjects were excluded due to too few SMS-answers (3/52 weeks and 17/52 weeks, respectively) leaving 30 subjects for the comparative analysis. The group consisted of 19 women and 11 men with a mean age of 45.9 (SD 10.5) years and a mean baseline pain intensity of 6.8 (SD 2.0).

#### Categorization of SMS-based trajectories

The two assessors agreed in 97, 93 and 77% of the cases in the intensity, variation and change domains, respectively. In case of disagreement, consensus was reached through discussion and all patients were classified in all three domains.

In the pain intensity domain, most (74%) were classified as ‘mild’ (0–3); in the variation domain, the most common category was ‘episodic’ (40%) and in the change pattern domain, ‘slow improvement’ was the most common (46%). Two examples of trajectories can be seen in Fig. 1. Individual ‘7’ is classified as ‘severe/fluctuating/gradual improvement’ and individual ‘18’ as ‘mild/episodic/gradual improvement’. All individual categorizations can be seen in Additional file 1.

#### Categorization of interview-based trajectories

Due to the explorative nature of the interviews, all patients could not necessarily be classified in all domains. In the variation domain all were classified, but seven patients could not be categorized in the pain intensity domain and six in the change pattern domain, because the relevant issues were not referred to during the interviews.

In the pain intensity domain, ‘moderate to severe’ was slightly more prevalent than ‘mild’ (52% vs. 48%); in the variation domain, the most common category was ‘episodic’ (37%) and in the change pattern domain, ‘rapid improvement’ was the most common (42%). All individual categorizations can be seen in Additional 1.

Typical quotes for each category within the three domains were:

- Intensity, intense: “*I got up and it got so extreme and then I couldn’t even walk anymore, …”* (ID 19)
- Intensity, mild: “*I feel a little pain in my back once in a while, but that doesn’t bother me much.* ”(ID 14)
- Variation, fluctuating: *“…, related to the scale we have been using at times, 1 – 10, it is about 3 – 5 approximately, it goes up and down.”* (ID 14)
- Variation, ongoing*: “I feel back pain every day more or less.”* (ID 8)
- Variation, episodic: “*I feel pain once in a while. And then, then I go see the chiropractor and do some exercises myself,…”* (ID 3)
- Variation, single episode: *“..what do I get, around three or four treatments, and then it is simply gone and there hasn’t been anything since – at all”* (ID 12)
- Change pattern, progressing: “*It has been sort of stable, lately I think it is getting worse”* (ID 23)
- Change pattern, ongoing: *“It has actually been stable. Well, but those pains are always there and.. what can you say… some days are worse than others, but I have back pain constantly”* (ID 24)
- Change pattern, gradual improvement: *“… I can feel it a little once in a while and so, but not as violently as it was in the beginning …”* (ID 29)
- Change pattern, rapid improvement: *“It came suddenly and it disappeared suddenly.”* (ID 9)

All categories for the individual patients are shown in Additional file 1.

### SMS-based vs. interview-based trajectory categorizations

Cross-tabulations between the categorizations based on interviews and the categorization based on SMS questions within the three domains: pain intensity, overall change patterns, and variation (frequency), respectively, are shown in Tables 1, 2, 3, 4.

**Table 1:** Interview-based versus SMS-based mean intensity of LBP measured on a scale from 0 to 10 over a 1 year course (all weeks included)

SMS-based	Interview-based		
	None to mild	Moderate to severe	Total
0–3	**35% (8)**	39% (9)	74% (17)
4–10	13% (3)	**13% (3)**	26% (6)
Total	48% (11)	52% (12)	100% (23)

Percentages of the population with absolute numbers in parentheses and agreement indicated in bold

**Table 2:** Post-hoc analysis: Interview-based versus SMS-based mean intensity of LBP over a 1 year course

SMS-based	Interview-based		
	None to mild	Moderate to severe	Total
0–3	**17% (4)**	17% (4)	35% (8)
4–10	30% (7)	**35% (8)**	65% (15)
Total	48% (11)	52% (12)	100% (23)

The SMS-based mean intensity calculated on basis of weeks with pain (pain-free weeks excluded). Percentages of the population with absolute numbers in parentheses and agreement indicated in bold

**Table 3:**
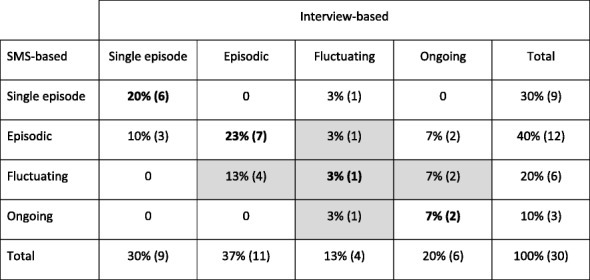
Interview-based versus SMS-based variation of LBP reported over a one-year course

Percentages of the population with absolute numbers in parentheses. Agreement indicated in bold. Agreement for the fluctuating pattern and the neighboring categories are shaded

**Table 4:**
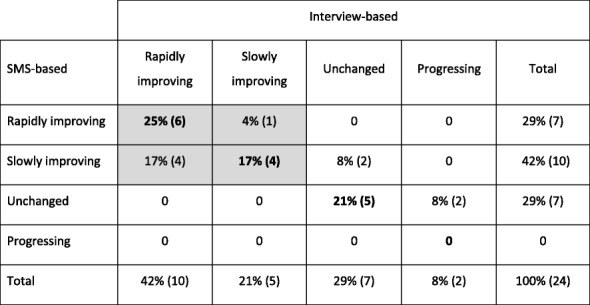
Overall change pattern of LBP reported over a one-year course

Percentages of the population with absolute numbers in parentheses and agreement indicated in bold. The two ‘improving’ categories are shaded

#### Intensity domain

The percentage of agreement for pain intensity was 48% and the major discrepancy (9 of the 12 discordant categorizations) was due to pain being rated higher in the interviews than from the SMS-track (Table 1). A possible reason for this was that six of those nine individuals were pain-free for most of the year and therefore had a low mean pain intensity, whereas the recalled pain was high, albeit only a brief period of time. However, restricting the calculation of mean pain intensity from SMS-track to pain episodes only in the post-hoc analysis, only improved agreement from 48 to 52%, i.e. agreement in one more case (Table 2).

There was no pattern detected of patients’ current pain level, as reported by SMS, influencing the interview-based categorization, i.e. high pain intensity at the end of follow-up resulting in recall of higher intensity pain by interviews than the averaged SMS-based pain intensity. Actually, of the nine individuals categorized with severe pain from interviews but mild pain from the SMS-track, five reported no pain and four reported 1 or 2 on the NRS the last 4 weeks.

#### Variation domain

For the variation domain, the agreement was 53% (Table 3). Of the 14 cases with discordant categorization, the most common disagreements related to the fluctuating pattern, which actually had only 3% agreement, but were categorized in neighboring categories (*n* = 8, shaded with grey in Table 3). The three individuals who were categorized with only a single episode by interview but ‘episodic’ by SMS-track, actually had 2–6 episodes according to the SMS-track, but they were all brief and of significantly less intensity and activity limitation than the initial episode (ID: 4, 10, 19). The two individuals who were considered to have ongoing pain based on interviews, but episodic based on SMS (ID: 8, 13), both had only one pain free episode lasting more than 1 month, and this was respectively 14 and 32 weeks prior to the interview. The last discrepancy relates to a patient who was categorized as ‘fluctuating’ by interview and ‘single episode’ by SMS (ID: 5). This individual had one minor episode 4 weeks after the initial episode, after which no pain was recorded. However, there were some missing answers which might have been pain episodes.

In general, no pattern was detected of patients rating themselves better or worse in interviews than by their SMS-measurements: eight patients were rated better in the interviews than by SMS-track, whereas the opposite was true for six patients.

#### Change domain

For the change domain, the agreement was 63% (Table 4). If the speed of improvement was ignored and consequently rapid and slow improvement combined into one category (indicated with grey shading in Table 4), the agreement would increase to 83%. However, it should be noted that six patients were not categorized by interviews because of uncertainty in this domain.

For five patients, the interview-based categorization indicated a worse trajectory than the SMS-track and for another four patients the opposite was the case. Ignoring the rate of improvement (rapidly or slowly), the corresponding figures were four and zero.

There were two patients rated as ‘unchanged’ on SMS-categorization and ‘progressing’ on interview-categorization. One of these was very constant in SMS reporting with pain intensities of 8 or 9 with only 1 week’s exception (ID: 21); the other had a very large variation across the whole year (ID: 23). Two patients were categorized as unchanged from interviews but showed a slowly improving pattern on the SMS-track. For one of these, the improvement was within the first 16 weeks, where after the SMS reports was constant for the rest of the year (ID: 14), and the other had an unchanged pattern of fluctuations for the first two thirds of the year, whereas the last third of the year had been good with pain reported only 1 week (ID: 18).

None of these disagreements could be explained by activity limitation, since this was either very low or followed the pattern of pain intensity, but the main discrepancies were within the first half of the year.

### Recovery status vs. trajectories

When asked directly, 15 responders thought they were over their back problems, nine did not and six were unsure. The 15 responders who answered “Yes” to being over their back problems were all categorized as either ‘single episode’ (*n* = 7) or ‘episodic’ (*n* = 8) in the variation-domain based on the SMS-trajectories.

The group who did *not* consider themselves to be over their back problems were mainly ‘fluctuating’ (*n* = 5) or ‘ongoing’ (*n* = 3), but one was classified as ‘episodic’ (ID = 8). However, this patient only had one pain free episode during the year and had rather high reports of pain the last 14 weeks, so based on the SMS-track that would not be considered as recovered either.

The trajectories from the six unsure patients showed that some of this uncertainty was explainable by recent recurrences or improvements without complete remissions. However, the uncertainty in two of the six seemed to be unrelated to the SMS-based trajectories.

## Discussion

This study explored whether researchers’ interpretation of frequent quantitative outcome measures (SMS track) was coherent with how patients describe their course and recovery and was a first attempt to compare quantitative and qualitative measures of a one-year course of LBP. Generally, the interviews supported the findings from the quantitative measures, but fluctuations were not a clear part of peoples recall and therefore difficult to define from interviews.

As expected, patients did not consider themselves as being recovered from their LBP when they were quantitatively defined as ‘fluctuating’ or ‘ongoing’. However, episodic pain appeared to have less impact, since eight individuals considered themselves recovered, despite being categorized as ‘episodic’ by SMS. This demonstrates that LBP is fluctuating in nature, and people with LBP patterns categorised as fluctuating had more severe and disabling LBP than those with episodic pain. Thus, it appears that it makes an important positive difference to people with LBP to have periods that are pain free. This is in line with previous observations [20].

We encountered no difficulties with assigning the SMS-based trajectories to the predefined categories, and all patients could be assigned to the variation category based on interviews, but there were difficulties with the domains of pain intensity and change pattern. One explanation could be a mismatch between the patients’ and researchers’ understanding of the concept of pain intensity. Regarding change patterns, the interviews should probably have explored the timeframe in more detail. Further research is needed to elaborate on this.

Although a substantial body of literature suggests that quantitative measures only reflect part of the patients’ experiences [25–27], the self-perceived recovery status seemed to be reflected quite well by the quantitative measures of pain intensity and days with pain in this study. Self-reported recovery status might be disproportionately influenced by present or recent pain status [28], but nevertheless our results indicate that a recovery question is a valid question for an end-point assessment, as has been showed previously [29]. However, the uncertainty in two of the six patients that were unsure of their recovery seemed to be unrelated to the SMS-based trajectories indicating that there are issues in the recovery perception of these participants which are not captured by the quantitative measures. This issue has been further investigated in a qualitative analysis of the same interviews, exploring the issues impacting on perceived recovery and showing that several participants had difficulties relating the concept of recovery to their experiences and some showing paradoxical scepticism, i.e. they remain skeptical of their backs during pain-free periods because they anticipate a new episode [9].

Overall, we did not detect a bias toward more or less pain intensity in the reporting from interviews compared with weekly SMS’s. This could however be due to inherent prompting in the design: when a patient recalls the pain, it is likely a reflection of the pain when it was present, whereas, the SMS-based categorization was a mean calculated across the whole year, including pain free episodes. However, the post-hoc analysis, ignoring pain free periods, did not improve the agreement substantially. Another theory is that the patients’ recollection of pain level is influenced by present pain level as has been reported previously [30] but such a pattern was not present in our data. In future studies, a distinction must be made between pain intensities during periods with pain, and pain intensity across the full follow-up period, and this should also be reflected clearly in interview guides.

With regard to variation in pain across the year, there was quite a lot of disagreement, indicating some weakness in the operational criteria for the distinction between categories within this domain. However, with the exception of three patients, all were categorized in the same or a neighboring category. Especially the definition of ‘episodic’ based on only one pain free episode during the course of a whole year might be questionable.

It appeared to be difficult to distinguish between slow and rapid improvement (more or less than 4 weeks), but if the rate of improvement is ignored, the change pattern domain had the best agreement of the three domains. The fact that the main discrepancies were within the first half of the year indicate that the first part of the year is either not recalled after 1 year [28] or is not considered important when patients describe their general change pattern retrospectively.

We believe, the combination of interviews with open-ended questions and the strictly quantitative data from the SMS-track provides important insight into the quality of both. However, the limited number of interviews limits the use of statistics, such as estimation of Kappa values for agreement. Furthermore, the fact that the patients have received weekly SMS-questions about pain and activity limitation is an inherent weakness in the design. First of all, the weekly focus on two LBP-related concepts (pain intensity and activity limitation) might have given these concepts a disproportionately large weight in the patients’ appraisal of their course at a subconscious level. Furthermore, the patients’ ability to recall their course of pain might have been attenuated by the constant answering of SMS-questions, requiring appraisal of the pain status every week which might explain why our results did not seem to be as hampered by recall bias as often reported in the literature [31]. Nevertheless, we believe the study illustrates that there is potential for improving outcome measures by working with trajectories rather than single time point measures. The SMS trajectories seem to reflect the patients’ experience to a large extent, and thus have the potential for an improved understanding of longitudinal change in fluctuating diseases like back pain. Furthermore, self-reported retrospective course appraisals at the end of a follow-up period might replace the usual one time-point measures, which are typically used today, providing more detailed knowledge about treatment effect and maybe a more informed prognosis for the future. If the possible trajectory patterns become better defined, it might be possible to ask patients to categorize themselves into such predefined categories, e.g. by presenting graphical trajectories similar to those shown in Fig. 1. Such an illustration could reflect the *course* rather than the *state* of pain and disability, and therefore might improve outcome measurements in future research. Such an approach has been tested by Dunn et al. using almost similar trajectory illustrations as in the present study, and they demonstrated acceptable face, criterion and construct validity [32]. However, in light of the disagreement encountered in this study, the trajectories could be refined further.

## Conclusion

This study shows that a real time quantitative measure (weekly SMS) and the patient’s retrospective appraisal do not fundamentally differ in their reflection of the one-year course of LBP.

As a first investigation into this area, these results are promising with regard to patient’s ability to retrospectively recall their one-year course of LBP and likewise, longitudinal quantitatively derived trajectories of LBP seem to reflect the lived experience of the patient to a large degree. Future research should focus on the optimal timeframe for recall, better description of distinct categories and the relative importance of the three domains.

## Additional file

Additional file 1: Individual categorizations by SMS-track and interview. Shows the categorization of each patient (random id-numbers) from both SMS-track and interviews. (DOCX 19 kb)

## Abbreviations

SMS: Short Message Service LBP Low Back Pain NRS Numeric Rating Scale
