# Supplementary material for: Contrasting real time quantitative measures (weekly SMS) to patients’ retrospective appraisal of their one-year’s course of low back pain; a probing mixed-methods study
Source: Chiropr Man Therap. 2019 Feb 26;27:12. doi: 10.1186/s12998-018-0222-y (PMC6390359; doi:10.1186/s12998-018-0222-y)
Supplement: Supplementary file 1 — Individual categorizations by SMS-track and interview. Shows the categorization of each patient (random id-numbers) from both SMS-track and interviews. (DOCX 19 kb) [file 12998_2018_222_MOESM1_ESM.docx]

Additional file 1: Individual categorizations by SMS-track and interview

|  | SMS-based trajectories | | | Interview based trajectories | | |  |
| --- | --- | --- | --- | --- | --- | --- | --- |
| ID | intensity | variation | change | intensity | variation | change | "recovery” |
| 1 | 1 | 3 | 1 | 2 | 2 | 2 | 9 |
| 2 | 1 | 1 | 2 | 2 | 1 | 9 | 1 |
| 3 | 1 | 2 | 3 | 1 | 2 | 9 | 1 |
| 4 | 1 | 2 | 1 | 2 | 1 | 1 | 1 |
| 5 | 1 | 1 | 1 | 2 | 3 | 1 | 1 |
| 6 | . | . | . | 1 | 2 | 3 | 1 |
| 7 | 2 | 3 | 2 | 2 | 2 | 2 | 0 |
| 8 | 1 | 2 | 3 | 2 | 4 | 3 | 0 |
| 9 | . | . | . | 9 | 1 | 1 | 1 |
| 10 | 1 | 2 | 2 | 1 | 1 | 2 | 9 |
| 11 | 1 | 2 | 2 | 1 | 2 | 2 | 1 |
| 12 | 1 | 1 | 1 | 9 | 1 | 1 | 1 |
| 13 | 1 | 2 | 4 | 9 | 4 | 9 | 9 |
| 14 | 2 | 3 | 2 | 1 | 3 | 3 | 0 |
| 15 | 2 | 3 | 3 | 9 | 2 | 9 | 0 |
| 16 | 1 | 2 | 1 | 1 | 2 | 1 | 1 |
| 17 | 1 | 1 | 3 | 9 | 1 | 9 | 1 |
| 18 | 1 | 2 | 2 | 1 | 2 | 3 | 9 |
| 19 | 1 | 2 | 2 | 2 | 1 | 1 | 1 |
| 20 | 1 | 1 | 1 | 9 | 1 | 1 | 1 |
| 21 | 2 | 4 | 3 | 9 | 4 | 4 | 0 |
| 22 | 2 | 4 | 3 | 2 | 4 | 3 | 0 |
| 23 | 2 | 3 | 3 | 1 | 4 | 4 | 0 |
| 24 | 1 | 4 | 3 | 2 | 3 | 3 | 0 |
| 25 | 1 | 1 | 1 | 2 | 1 | 1 | 1 |
| 26 | 1 | 2 | 2 | 1 | 2 | 1 | 1 |
| 27 | 1 | 2 | 3 | 1 | 3 | 3 | 1 |
| 28 | 2 | 3 | 3 | 2 | 4 | 3 | 0 |
| 29 | 2 | 3 | 2 | 1 | 2 | 2 | 9 |
| 30 | 1 | 2 | 2 | 9 | 2 | 9 | 9 |
| 31 | 1 | 2 | 2 | 1 | 2 | 1 | 1 |
| 32 | 1 | 1 | 2 | 2 | 1 | 1 | 1 |

Intensity Variation Change pattern Recovery
1: Mild/Minor pain/Recovery 1: Single episode 1: Rapid improvement 0: No
2: Severe/moderate pain 2: Episodic 2: Gradual improvement 1: Yes
9: Unsure 3: Fluctuating 3: Unchanged 9: Unsure

4: Ongoing 4: Progressing

9: Unsure 9: Unsure
